# Behaviour and Psychopathology in Preschool Children with William Syndrome and the Effects of Age, Sex and Cognition

**DOI:** 10.1007/s10803-024-06530-z

**Published:** 2024-10-07

**Authors:** Daniel Miezah, Melanie Porter, Jennifer Batchelor, Adriana Rossi, Jessica Reeve

**Affiliations:** School of Psychological Sciences, Macquarie University, Herring Road, North Ryde, Sydney, NSW 2109 Australia

**Keywords:** Williams syndrome, Cognitive abilities, Psychopathology, Behaviour impairments

## Abstract

The current study compared the prevalence of cognitive and psychopathological impairments among 24 preschool children with Williams syndrome (WS) (aged 2.20 to 5.97 years) and 53 controls without WS and screened for developmental or psychological diagnoses (aged 2.21 to 5.89 years) matched on chronological age and sex distribution. Associations between sex, chronological age, early development and psychopathology were also investigated. The Child Behavior Checklist—Preschool Version (CBCL) and the Mullen Scales of Early Learning were administered. Higher reported rates of Attention Problems, Emotional Reactivity, Somatic Complaints, Withdrawal, Affective Problems and Total Problems were found in preschool children with WS. There were no significant group differences in prevalence rates of all other CBCL domains. Attention Problems were the most prevalent psychopathology in preschool children with WS (33% falling in the clinically significant range), followed by Affective Problems (29% in clinically significant range), then Anxiety Problems (17%) or Attention Deficit/Hyperactivity Problems (17%). Among children without WS, the highest prevalence rates of psychopathology were for Attention Problems (4% falling in the clinically significant range), Aggressive Behaviour (4%), Sleep Problems (4%) and Oppositional Defiant Problems (4%). There were no significant associations between sex or chronological age and CBCL-reported psychopathology for either group. In addition, there were no significant relationships between CBCL ratings and verbal ability, nonverbal ability or overall developmental level in either group. Findings highlight variations in the pattern of psychopathology among preschool children with WS compared to those without WS, which needs to be considered in clinical management and future research.

Williams Syndrome (WS), also is sometimes referred to as Williams-Beuren Syndrome, is a rare neurodevelopmental condition resulting from a copy number variant involving f 25 to 28 genes (a 1.5- to 1.8-Mb 7q11.23 deletion) (Blomberg et al., 2006; Korenberg et al., 2000). The estimated prevalence of WS is 1:7,500 to 1:20,000, occurring equally across males and females (Kozel et al., 2021). Common features of WS include: distinct facial features, hyper-sociable personality, intellectual disability and more specific cognitive impairments (e.g., inattention, poor spatial abilities), social problems and behavioural and psychological impairments (e.g., hyperactivity, behavioural disinhibition, anxiety and depression) (Kozel et al., 2021). While there has been considerable research on behaviour and psychopathology in older children, adolescents and adults with WS, very little research has been undertaken on young children with WS. This includes a lack of developmental profiling. Investigations on behaviour and psychopathology and early development in young children with WS is essential to aid in early identification, management and prevention.

## Behaviour and Psychopathology Profiles in WS

There is a paucity of studies examining behaviour problems and psychopathology in preschool children with WS. While most of these studies have investigated specific aspects of the behaviour and psychopathological profile (Dodd et al., 2010; Jones et al., 2000; Klein-Tasman et al., 2007, 2009; Lincoln et al., 2007; Mervis et al., 2003), a select few studies have examined a broad range of problems using comprehensive measures (Axelsson et al., 2013; Braga et al., 2018; Hahn et al., 2014; Klein‐Tasman & Lee, 2017; Papaeliou et al., 2012). Studies report on the following difficulties in pre-schoolers with WS: attention problems, emotional regulation impairments, aggression, anxiety, sleep problems and more general internalising and externalising difficulties (Axelsson et al., 2013; Braga et al., 2018; Hahn et al., 2014; Klein‐Tasman & Lee, 2017; Papaeliou et al., 2012). Axelsson et al. (2013) utilised the Child Behaviour Checklist 1.5–5 (CBCL 1.5–5) in 14 WS children (aged 1.54 to 4.01 years) and 14 Controls without a diagnosed developmental condition (aged 1.50 to 3.96 years) and reported that significantly higher numbers of children with WS were classified in the borderline/clinical impairment range on Emotionally Reactive, Sleep Problems and Attention Problems compared to control children without WS. In a similar study, Papaeliou et al. (2012) found that young children with WS (*n* = 20, mean age = 5.12 years) had significantly higher mean raw scores than both children with Down syndrome (DS) (*n* = 20, mean age = 5.40 years) and controls with no diagnosed developmental condition (*n* = 20, mean age = 2.51 years) in Emotional Problems, Anxiety/Depression, Internalising Problems, and Total Problems as measured by the CBCL (1.5–5). The wide differences between the mean chronological age of the groups is a limitation, although they were matched on mental age. Commonly, the difference in prevalence rates (for clinically elevated levels of difficulty) between preschool children with WS and controls is not reported. In addition, findings on the most prevalent areas of difficulty in preschool children with WS are inconsistent. For example, using the CBCL (1.5–5) Klein‐Tasman and Lee (2017) reported attention problems as the most prevalent area of difficulty among 35 pre-school children with WS (aged 2 to 6 years), whereas Braga et al. (2018) reported the highest prevalence on the anxiety/depression subscale in 8 children with WS (aged 4 to 6 years) using both the CBCL (1.5–5) and CBCL (6–18). These studies examined average levels of impairment and did not explore individual variability in any detail.

Sarimski (1997) demonstrated within-syndrome variability in the behavioural phenotype of pre-school children with WS (*n* = 16, age range = 1 to 5 years) using the Verhaltensfragebogen ftir Vorschulkinder which is a German test for measuring social competence and behavioural problems (oppositional-aggressive behaviours, hyperactive behaviours and emotional problems) in preschool children. Similarly, Porter et al. (2009) indicated variability in reported behaviour and psychological problems of individuals with WS across the lifespan (aged 6–48 years, *n* = 31) using the Child Behavior Checklist (CBCL 6–18; Achenbach & Rescorla, 2001). Sex and specific cognitive abilities made independent significant contributions to the variance. For example, females with WS showed significantly more externalising problems than males.

## Early Cognitive/Developmental Profiles in WS

There is a substantial literature base on the cognitive strengths and weaknesses and the intellectual profile(s) of older children, adolescents and adults with WS (e.g., see Kozel et al., 2021 and Martens et al., 2008 for reviews). Intellectually, individuals with WS, on average, fall in the mild to moderate range (Martens et al., 2008), and verbal and nonverbal intellectual discrepancies can vary in magnitude, direction and significance.

Overall, it is indicated that approximately 75% of those with WS will have an intellectual disability, but there is also heterogeneity in overall levels of intellect, in intellectual profiles, and in more specific cognitive strengths and weaknesses.

There are very few studies looking at early developmental profiles in WS. One study of 16 children with WS aged 3 to 65 months found that motor skills were significantly lower than both cognitive and language skills, on average, on the Bayley Scales of Infant and Toddler Development, Third Edition (Kirchner et al., 2016). However, individual profiles were not discussed.

## Effects of Chronological Age, Sex and Cognition on Behaviour and Psychological Profiles in WS

Findings from studies examining the effects of chronological age on behaviour and emotional problems in WS are inconsistent. Some studies using mixed samples of older children, adolescents and adults with WS have shown that the prevalence of Generalised Anxiety Disorder (GAD) and depressive disorders was significantly higher in older compared to younger individuals with WS (Dodd & Porter, 2009; Leyfer et al., 2006). (Klein-Tasman et al., 2015; Porter et al., 2009; Riby et al., 2014). Also, in a diagnostic study, the prevalence of ADHD was significantly higher for children than adults with WS (Dodd & Porter, 2009).

Similarly, albeit focusing on psychopathological symptomology rather than diagnoses, Klein-Tasman et al. (2015) found a significant, positive relationship between chronological age and ratings on the anxious/depressed subscale of the child behavior checklist (CBCL 6–18; Achenbach & Rescorla, 2001) in 52 children with WS aged 6 to 17 years. Klein-Tasman et al. (2015) found no significant relationship between chronological age and parent reports of behaviour difficulties. Moreover, some studies have reported no effects of chronological age on the prevalence of behaviour and emotional problems among mixed samples of older children, adolescents and adults with WS.

Only one study has examined the relationship between chronological age and behaviour problems in preschool children with WS (Klein‐Tasman & Lee, 2017). Klein‐Tasman and Lee (2017) found that age was positively related to only Anxiety Problems in WS young children (aged 2 to 6 years) when teacher ratings of the (CBCL) were used. In contrast, they found no significant relationships between chronological age and parent ratings of behaviour problems.

Studies on the effects of sex on behaviour and emotional problems in WS have reported mixed findings. Some studies report no sex differences (Dodd & Porter, 2009; Kennedy et al., 2006; Klein-Tasman et al., 2015; Klein‐Tasman & Lee, 2017; Leyfer et al., 2006; Osório et al., 2017; Porter et al., 2009; Riby et al., 2014). However, other studies have found sex differences in some aspects of behaviour and psychological difficulties in WS (Blomberg et al., 2006; Dykens, 2003; Gosch & Pankau, 1997; Klein‐Tasman & Lee, 2017; Porter et al., 2009). For example, Porter et al. (2009) found that females displayed significantly higher Externalising, Affective, Somatic Problems and Conduct Problems than males with WS; the opposite pattern to that typically reported in the general population. In addition, some studies have reported that specific fears/ phobias were more prevalent in females than in males with WS (Blomberg et al., 2006; Dykens, 2003). In a preschool study, Klein‐Tasman and Lee (2017) reported that WS preschool-aged males scored higher than females on parent ratings of Affective Problems using the Child Behaviour Checklist Preschool Form (CBCL). In addition, no significant sex differences were noted on the remaining CBCL subscales. There seems to be a paucity of studies examining sex differences in reported problems in preschool children with WS and more generally. Moreover, in most studies (across the lifespan) males and females with WS were not matched on potential confounding variables such as chronological age, verbal ability, nonverbal ability, and overall cognitive ability before examining sex differences.

Results from studies which examined the relationship between cognition and behaviour problems/psychopathology in WS are also inconsistent. Some studies have found no significant relationship between cognition and behaviour problems/psychopathology in WS samples across both diagnostic studies and those utilising symptom screeners (Dodd & Porter, 2009; Klein‐Tasman & Lee, 2017; Leyfer et al., 2006; Porter et al., 2009; Riby et al., 2014; Stinton et al., 2010; Woodruff‐Borden, Kistler, Henderson, Crawford, & Mervis, 2010). Other studies have shown significant, negative relationships between cognition and behaviour problems/psychopathology in WS utilising symptom screeners (Klein-Tasman et al., 2015; Osório et al., 2017; Porter et al., 2009). For example, Osório et al. (2017) found a significant, negative relationship between rule-breaking behaviour and general IQ, verbal IQ and performance IQ in young children with WS (*n* = 8 aged = 7 to 12 years), while there were significant, negative associations between internalising problems (Anxious/Depressed or Withdrawn/Depressed) and general IQ, verbal IQ and performance IQ in WS adolescents (*n* = 17 aged = 13 to 18 years). In this same study, Social Problems were significantly and negatively related to verbal IQ in adolescents with WS. This study suggests that the pattern of relationships between cognition and behaviour problems/psychopathology in WS is moderated by age. In a similar study, Klein-Tasman et al. (2015) reported a significant, negative relationship between general level of intellectual functioning and the Thought Problems scale on the CBCL in individuals with WS (*n* = 52, aged 6 to 17 years).

In contrast to findings above, significant, positive relationships have been reported between cognition and behaviour problems/psychopathology in WS (Braga et al., 2018; Ng et al., 2014; Porter et al., 2009). Braga et al. (2018) reported a positive relationship between language impairment (Denver Developmental Screening Test-II) and aggressive behaviour problems (Behaviour Problems Inventory) in WS preschool children (*n* = 8, aged range = 4 to 6 years). The use of a smaller sample size of WS preschool children (*n* = 8) was a limitation in this study (Braga et al., 2018).

### The Current Study

In light of the above, the aims of the current study were to, firstly, investigate the prevalence of behavioural impairments and psychopathological symptoms in preschool children with and without WS, the latter being the control sample. The second aim was to examine cognitive functioning (Nonverbal, Verbal and overall DQ) in these same groups, and the final aim was to examine the effects of sex, chronological age, and developmental ability (Nonverbal, Verbal and overall DQ) on behavioural impairments and psychopathology in each group. There were no specific hypotheses made given the sparse research available on preschool children with WS, however, psychological and behavioural findings were predicted to more generally follow findings within the adult literature, especially in relation to psychopathology and cognitive profiles.

## Method

### Participants

The study included 24 preschool children with WS (10 males, 14 females) between the ages of 2.20 to 5.97 years (males: *M* = 4.19 years, *SD* = 1.17; females: *M* = 4.06 years, *SD* = 1.25) and a control group without WS and without a diagnosed developmental or psychological condition involving 53 individuals (28 males, 25 females) between the ages of 2.21 to 5.89 years (males: *M* = 3.79 years, *SD* = 0.97; females: *M* = 4.11 years, *SD* = 1.16). Demographic information is shown in Table 1. The children with WS and controls without WS were matched on chronological age (*t*(75) = 0.64, *p* = 0.52) and sex distribution (Fisher’s exact test, *p* = 0.46). Individuals with WS were recruited through Williams Syndrome Australia Limited and the New Zealand Williams Syndrome Association. WS diagnosis was confirmed using the Fluorescent in Situ Hybridization (FISH) test (Fryssira et al., 1997). Control children were recruited through the Macquarie University’s Neuronauts Brain Science Club. Early cognitive ability of all children (those with WS and controls without WS) was assessed using the Mullen Scales of Early Learning (MSEL; Mullen, 1995). Children without WS were screened for a history of developmental delay, intellectual impairment, learning disorder, neurological condition, psychological disorder, or major sensory impairment. No control child was excluded from the study based on the above criteria. Children with WS were screened for a history of psychological, neurodevelopmental, and/or neurological conditions unrelated to their WS (e.g., peri-natal hypoxic brain injury, acquired brain injury, etc.), and major sensory impairments that could impact on their ability to perform the research tasks. No child with WS was excluded from the study based on the above criteria.

**Table 1:** Descriptive Statistics for WS preschool children and TD controls

Variable	WS sample (*n* = 24)	Control group (*n* = 50 for Mullens DQs and *n* = 53 for CBCL)
	Mean (*SD*) [range]	Mean (*SD*) [range]
Chronological age (years)	4.12 (1.20) [2.20–5.97]	3.94 (1.06) [2.21–5.89]
DQ Scores on the Mullens		
Overall	55.11 (13.25) [28.62–73.36]	104.18 (12.79) [69.67–134.03]
Verbal	54.90 (15.69) [23.65–79.48]	105.77 (14.77) [64.81–141.88]
Nonverbal	55.32 (13.23) [31.49–77.38]	102.59 (13.17) [68.92–135.79
CBCL scale *T* scores		
Internalising problems	57.63 (9.74) [33.00–**71.00**]	42.60 (9.53) [29.00–61.00]
Emotionally reactive	59.33 (7.30) [50.00–**73.00**]	51.84 (3.86) [50.00–65.00]
Anxious/depressed	54.75 (4.59) [50.00–66.00]	51.26 (2.50) [50.00–59.00]
Somatic complaints	58.71 (7.94) [50.00-** 76.00**]	52.51(4.50) [50.00–65.00]
Withdrawn	60.08 (6.33) [50.00–**70.00**]	51.57 (3.11) [50.00–63.00]
Externalising problems	56.83 (11.14) [37.00–**80.00**]	42.94 (10.28) [28.00–**76.00**]
Attention problems	64.33 (8.88) [51.00–**77.00**]	51.89 (4.78) [50.00–**77.00**]
Aggressive behavior	57.33 (8.06) [50.00–**79.00**]	51.92 (4.81) [50.00–**72.00**]
Sleep problems	56.79 (6.85) [50.00–**76.00**]	53.51 (5.60) [50.00–**76.00**]
Total problems	57.79 (11.47) [37.00–**77.00**]	42.55 (9.81) [28.00–**68.00**]
Affective problems	62.58 (8.10) [50.00–**77.00**]	52.68 (4.09) [50.00–67.00]
Anxiety problems	57.42 (8.34) [50.00–**73.00**]	51.94 (4.05) [50.00–**70.00**]
Pervasive developmental problems	60.67 (6.57) [50.00–**72.00**]	52.21 (3.99) [50.00–66.00]
Attention deficit/hyperact problems	59.42 (8.34) [50.00–**76.00**]	51.89 (4.49) [50.00–**76.00**]
Oppositional defiant problems	56.71 (7.78) [50.00–**80.00**]	52.57 (5.09) [50.00–**73.00**]

Bold typeface indicates clinically significant scores

*CBCL* child behavior checklist, *DQ* developmental quotient

### Measures

#### Child Behavior Checklist 1.5–5 (CBCL)

The CBCL (Achenbach & Rescorla, 2000) was used to assess psychopathology symptoms and behavioural impairments among the preschool sample. The CBCL was completed by the parents/caregivers of each child (with or without WS) and were asked to rate each of the 100 items describing their child’s behaviour now or within the past two months. The CBCL has been used in previous studies on preschool children with WS (e.g., Braga et al., 2018; Klein‐Tasman & Lee, 2017), and older children and adults with WS (e.g., Klein-Tasman et al., 2015; Porter et al., 2009), and on children with other developmental disabilities (e.g., Autism Spectrum Disorder and Down syndrome; e.g., Guralnick et al., 2011; Pandolfi, Magyar, & Dill, 2009; Sikora et al., 2008). Raw scores are converted to *T* scores (*M* = 50, *SD* = 10) and were used for all analysis in the present study.

#### Mullen Scales of Early Learning (MSEL)

The MSEL (Mullen, 1995) is used for assessing cognitive development in infants and preschool-age children from birth to 68 months (Mullen, 1995). The test–retest reliability of the MSEL ranges between 0.71 and 0.96 (Mullen, 1995).

In line with previous studies with young children with neurodevelopmental disorders, including WS (e.g., Kazzi et al., 2021), three Developmental Quotient (DQ) scores were manually calculated as many of the young WS children were at floor (standard *T* score of 20) on each of the individual core subtests. DQ scores were calculated for each MSEL subtest utilising the formula: DQ = age equivalent scores / chronological age × 100. Nonverbal DQ was calculated by averaging the DQ scores for Fine Motor and Visual Reception, Verbal DQ by averaging Receptive Language and Expressive Language scores, and Overall DQ by averaging all four core DQ scores. For further details, see Bishop et al. (2011), Farmer et al., (2016), and Richler et al. (2007).

#### Procedure and Scoring

Ethics approval for this study was gained from the Macquarie University Human Research Ethics Committee (reference numbers: 5200900071 and 52,021,913,524,613). The MSEL was administered to participants according to the standardised instructions (Mullen, 1995) by the last author (JR), a clinical neuropsychologist in training who was trained and had substantial expertise in test administration and in gaining rapport with young children. The CBCL was completed by parents/caregivers on the same day as the cognitive testing. Both the MSEL and CBCL were scored by hand and then checked with their respective computer scoring programs (Achenbach & Rescorla, 2000; Mullen, 1995). The order of MSEL items were randomised to avoid any systematic effects on tasks and to maintain children’s motivation, as per the standardised instructions (Mullen, 1995).

### Analytic Approach

Data analysis was conducted using the IBM SPSS Statistics version 25. Pearson correlation coefficients were utilised to examine the relationships between chronological age, cognitive ability (Nonverbal DQ, Verbal DQ, and Overall DQ), and behaviour impairment/psychopathology. Fisher’s exact tests were used for testing differences in prevalence of behavioural impairments and psychopathology symptoms between preschool children with and without WS. MANOVA was then used to test differences in levels of behavioural impairments and psychopathology symptoms between groups and to examine sex differences within both groups. Boxplots were created to illustrate the distribution of CBCL *T* scores for both WS and control children. *T* scores CBCL and DQ scores (MSEL) were utilised for all analysis in the present study. Missing data (n = 3 for MSEL) was treated as missing. These assessments were not obtained due to practical reasons (illness, unavailability). Bonferroni corrections were used for correlation analyses (p < 0.01) to determine statistical significance in order to avoid a type 1 error.⁠⁠⁠ Bonferroni adjustments were not made for any other analysis given the small sample size and to control for both type 1 and type 2 errors, here the alpha level was set at 0.05 level of significance.

## Results

### Internal Consistency on the CBCL

Cronbach alpha across empirically based and DSM scales was sound: 0.943 for the entire sample, 0.915 for the WS sample and 0.932 for the control sample.

### Differences Between Groups in Levels of Behavioural Impairments and Psychopathological Symptoms

Table 1 shows descriptive statistics for CBCL scales. Using the Wilks’s statistic, there was a statistically significant effect of CBCL behavioural impairments and psychopathological symptoms between WS and control samples, Λ = 0.36, *F* (15, 61) = 7.06, *p* = 0.000. When the results for each CBCL scale were considered separately, the WS group had a significantly higher mean *T* score relative to control controls on the following CBCL scales: Emotionally Reactive (*F* (1, 75) = 34.59, *p* = 0.000), Anxious/Depressed (*F* (1, 75) = 18.56, *p* = 0.000, partial eta squared = 0.19), Somatic Complaints (*F* (1, 75) = 18.98, *p* = 0.000), Withdrawn (*F* (1, 75) = 62.99, *p* = 0.000,), Attention Problems (*F* (1, 75) = 63.79, *p* = 0.000), Aggressive Behaviour (*F* (1, 75) = 13.40, *p* = 0.000), Internalising Problems (*F* (1, 75) = 40.43, *p* = 0.000), Externalising Problems (*F* (1, 75) = 28.62, *p* = 0.000), Total Problems (*F* (1, 75) = 35.84, *p* = 0.000), Affective Problems (*F* (1, 75) = 50.94, *p* = 0.000), Anxiety Problems (*F* (1, 75) = 15.13, *p* = 0.000), Pervasive Developmental Problems (*F* (1, 75) = 48.60, *p* = 0.000), Attention Deficit/Hyperactivity Problems (*F* (1, 75) = 26.49, *p* = 0.000), and Sleep Problems (*F* (1, 75) = 4.91, *p* = 0.030).

### Behaviour Impairments/Psychopathology in Preschool Children With WS and Controls

**Fig. 1:**
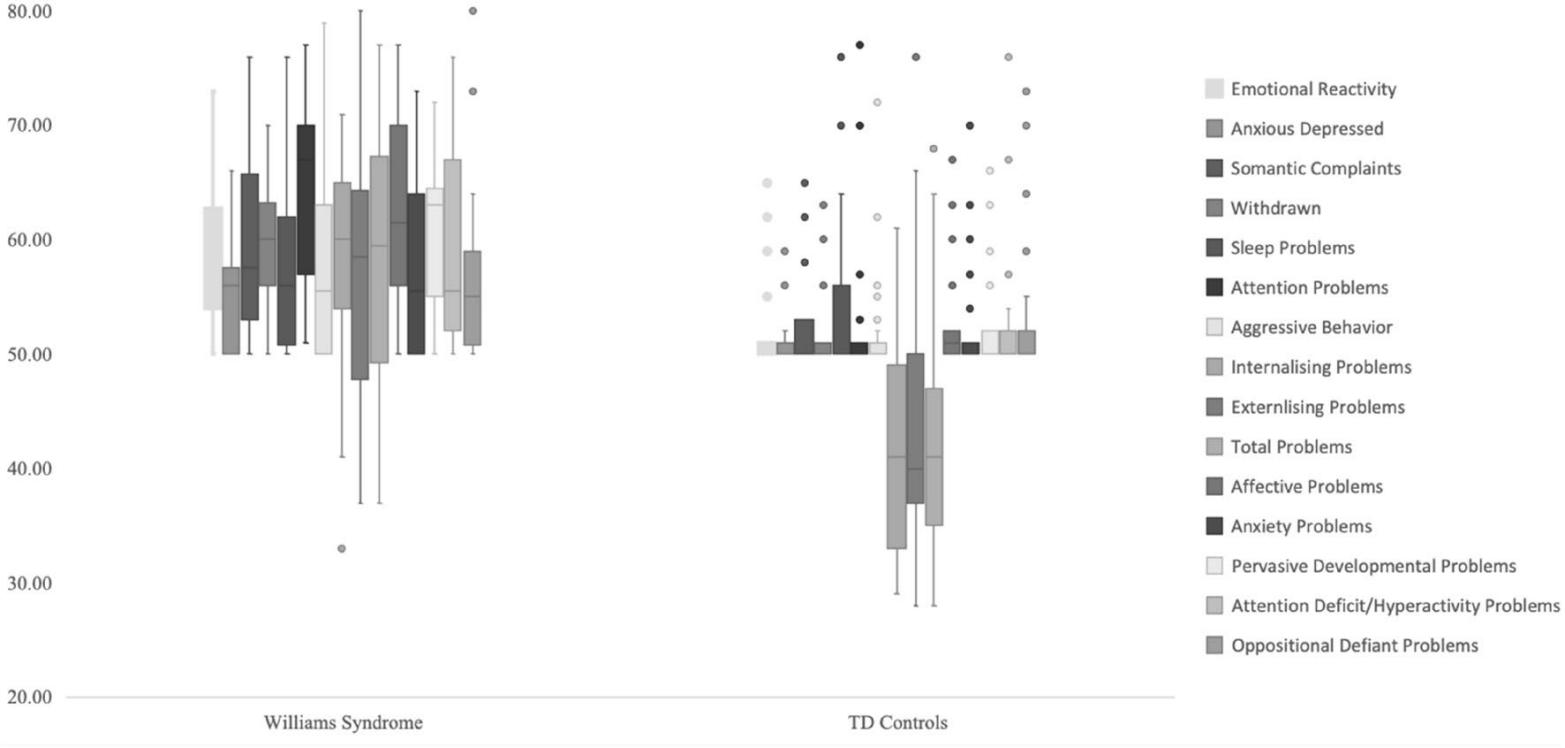
Distribution of *T* scores on behaviour impairments/psychopathology in WS and control *groups*. Variables are in the same order (left to right) as the variable list on the RHS

Figure 1 shows boxplots representing the distribution of CBCL *T* scores (*M* = 50) for preschool children with WS and controls without WS, respectively. Figure 1 demonstrates variability in the severity of behavioural impairments and psychopathology symptoms in preschool children with WS. Figure 1 indicates variability across both WS and control samples, with many scales ranging from within normal limits to clinically elevated for both groups.

### Prevalence of Clinically Elevated Behavioural Impairments and Psychopathological Symptoms in Preschool Children With and Without WS

To further investigate CBCL ratings, Table 2 shows frequencies and percentages of clinically elevated CBCL behavioural impairment and psychopathology scale *T* scores (i.e., clinical elevated range: *T* score > 70 for empirically based scores and DSM oriented scales; *T* score > 63 for Internalising Problems, Externalising Problems, and Total Problems scores) for preschool children with and without WS. Of note, the most prevalent reported problem for preschool children with WS was Attention Problems, followed by Affective Problems. In contrast, Sleep Problems, Pervasive Developmental Problems and Anxious/Depressed were rated lowest among the children with WS. Among the control group, the most commonly reported and clinically elevated areas of difficulty were Attention Problems, Aggressive Behaviour, Sleep Problems and Oppositional Defiant Problems.

**Table 2:** Prevalence of clinically elevated cbcl behavioural and psychopathological scales for in WS preschool children and TD controls (T scores)

CBCL Scales	WS (*n* = 24) No. (%)	Controls (*n* = 53) No. (%)	Fisher exact *p-*value comparing prevalence rates
Internalising problems	2 (8)	0 (0)	.094
Emotionally reactive	3 (13)	0 (0)	.027*
Anxious/depressed	0 (0)	0 (0)	1.000
Somatic complaints	3 (13)	0 (0)	.027*
Withdrawn	3 (13)	0 (0)	.027*
Externalising problems	2 (8)	1 (2)	.227
Attention problems	8 (33)	2 (4)	.001**
Aggressive behavior	2 (8)	2 (4)	.584
Sleep problems	1 (4)	2 (4)	1.000
Total problems	4 (17)	0 (0)	.007**
Affective problems	7 (29)	0 (0)	.000**
Anxiety problems	4 (17)	1 (2)	.030*
Pervasive developmental problems	1 (4)	0 (0)	.311
Attention deficit/hyperactivity problems	4 (17)	1 (2)	.030*
Oppositional defiant problems	2 (8)	2 (4)	.584

Clinical elevated range for empirically based scores and DSM oriented scales = *T* score > 70. Clinical elevated range for Internalising Problems, Externalising Problems, and Total Problems scales = *T* score > 63

** p < .01, and * p < .05

We found statistically significant and higher prevalence rates for the WS group relative to the Control group on following scales: Emotionally; Somatic Complaints; Withdrawn; Attention Problems; Total Problems; Affective Problems; Anxiety Problems and Attention Deficit/Hyperactivity Problems (See Table 2).

### Prevalence of Developmental Delay and Developmental Profiles in WS Preschool Children and Controls Without WS

Table 1 shows MSEL scores for each group separately, including overall DQ, verbal DQ and nonverbal DQ. As can be seen in Table 1, on average, children with WS performed in the range of a mild to moderate developmental delay, which is in keeping with the literature on WS (Martens, et al., 2008). All individuals with WS displayed a developmental delay, overall, all children with WS showed a delay in terms of both motor and verbal development. This is compared to three controls showing an overall developmental delay (6%), one control showing a motor delay (2%) and three showing a verbal delay (6%).

In terms of substantial or significant discrepancies across verbal and motor development (with a substantial discrepancy defined as one standard deviation or more difference in DQs across verbal and nonverbal domains (Mullen, 1995), three children with WS (4%) showed a verbal DQ greater than their nonverbal DQ, and three children (4%) showed the opposite pattern. The majority of WS children (92%) showed similar DQs across verbal and nonverbal domains. For controls, five children (7%) showed a verbal DQ greater than their nonverbal DQ, and eight children (11%) showed the opposite pattern. The majority of control children (82%) showed similar DQs across verbal and nonverbal domains. With the more conservative approach of 1.5 standard deviations, no child with WS showed such a large discrepancy, and two controls (4%) showed higher nonverbal DQ compared to verbal DQ.

### The Effects of Sex, Chronological Age, and Developmental Ability on CBCL Behavioural and Psychopathological Scales for Each GROUP

#### Sex Differences in Behavioural Impairments/Psychopathological Symptoms for Pre-Schoolers With and Without WS

Using the Wilks’s statistic, there was not a statistically significant effect between sex and CBCL behavioural impairments and psychopathological symptoms among the WS preschool children, Λ = 0.35, *F* (8, 15) = 0.97, *p* = 0.540, or the control group, Λ = 0.75, *F* (15, 37) = 0.82, *p* = 0.646.

#### Correlations Between Chronological Age, Developmental Ability, and CBCL Behavioural and Psychopathological Scales for Preschool Children With and Without WS

Table 3 shows Pearson’s correlations between chronological age, developmental ability, and CBCL behaviour and psychopathology scales in WS preschool children. No significant relationships were found between CBCL ratings and chronological age or developmental ability (Nonverbal DQ, Verbal DQ, or Overall DQ) for the WS or the control group (Table 4).

**Table 3:** Correlations between chronological age, cognitive ability and CBCL behavioural and psychopathological scales in WS preschool children (T scores)

CBCL scales	CA	Overall DQ	Nonverbal DQ	Verbal DQ
Internalising problems	−.12	.16	.25	.06
Emotionally reactive	.12	.39	.41	.31
Anxious/depressed	.03	.13	.23	.03
Somatic complaints	−.02	.22	.44	.01
Withdrawn	−.13	−.40	−.44	−.30
Externalising problems	.09	.39	.44	.29
Attention problems	.19	.04	.04	.03
Aggressive behavior	.05	.36	.44	.24
Sleep problems	−.20	.38	.52	.19
Total problems	−.09	.27	.45	.07
Affective problems	−.01	.17	.22	.10
Anxiety problems	.06	.39	.47	.26
Pervasive developmental problems	−.09	−.19	−.10	−.23
Attention deficit/hyperactivity	.03	.16	.24	.06
Oppositional defiant problems	.19	.32	.35	.24

No correlations were significant with the Bonferroni adjustment of p < .01

*CA *chronological age, *DQ *developmental quotient

**Table 4:** Correlations between chronological age, cognitive ability and CBCL Behavioural and Psychopathological Scales in TD control group (T scores)

CBCL scales	CA	Overall DQ	Nonverbal DQ	Verbal DQ
Internalising problems	−.17	−.08	−.09	−.05
Emotionally reactive	−.08	.01	−.08	.09
Anxious/depressed	−.12	−.17	−.15	−.17
Somatic complaints	−.26	−.08	−.03	−.10
Withdrawn	−.16	−.27	−.23	−.26
Externalising problems	−.42	−.09	−.09	−.07
Attention problems	−.34	−.09	−.12	−.05
Aggressive behavior	−.18	−.15	−.19	−.10
Sleep problems	−.39	.11	.10	.10
Total problems	−.38	−.06	−.05	−.05
Affective problems	−.39	.03	.04	.02
Anxiety problems	−.14	−.14	−.12	−.13
Pervasive developmental problems	−.35	−.17	−.06	−.25
Attention deficit/hyperactivity	−.35	−.09	−.11	−.06
Oppositional defiant problems	−.26	−.19	−.23	−.14

No correlations were significant with the Bonferroni adjustment of p < .01

*CA* chronological age, *DQ* developmental quotient

## Discussion

In relation to the study aims, findings revealed variability in parent ratings of behaviour difficulties and psychopathology in our WS cohort, consistent with previous literature (e.g., Porter et al., 2009; Sarimski, 1997). No such variability was found for controls without WS. Also, consistent with previous research (Axelsson et al., 2013; Braga et al., 2018; Hahn et al., 2014; Klein‐Tasman & Lee, 2017; Papaeliou et al., 2012), the most commonly reported areas of difficulty in preschoolers with WS were: attention problems, affective problems and anxiety. Our prevalence of 33% in relation to attention problems is not as high as the 76% reported in Osório et al. (2017), possibly because their prevalence estimate included both clinical and borderline scores,whereas the present study focused on scores in the clinical range. Osório et al. (2017)’s use of older WS children and adolescents (7 to 18 years) compared to WS pre-schoolers in the current study may also partially explain the discrepancies.

Moreover, we found that preschoolers with WS had significantly higher *T* scores on Attention and Affective Problems, Emotional Reactivity, Somatic Complaints. Withdrawn, Attention Deficit/Hyperactivity and Total Problems than controls without WS. Papaeliou et al. (2012) found that WS pre-schoolers had significantly higher mean raw scores than controls without WS in these areas on the same measure.

In relation to the second aim, in line with Kozel et al. (2021), we found that all preschoolers with WS displayed developmental delay on the Mullen Scales of Early Leaning, and the majority showed similar levels of delay across motor and verbal domains. The average level of delay was mild to moderate, consistent with the IQ literature in WS (Kozel, et al., 2021). In contrast, developmental delay was uncommon in controls without WS.

In relation to the third aim, no significant sex differences in behaviour impairments/psychopathology were obtained in our cohort. This finding is consistent with select studies involving older children, adolescents and adults with WS (Dodd & Porter, 2009; Kennedy et al., 2006; Leyfer et al., 2006; Osório et al., 2017; Riby et al., 2014) and a study of pre-schoolers with WS (Klein‐Tasman & Lee, 2017), but is inconsistent with other studies (Blomberg et al., 2006; Dykens, 2003; Klein‐Tasman & Lee, 2017; Porter et al., 2009). Inconsistencies may be due to the different age ranges of WS samples and to methodological differences across studies. Moreover, in most studies WS males and females were not matched on potential confounding variables such as chronological age, verbal ability, nonverbal ability, and overall cognitive ability before examining sex differences.

Our results showed no significant relationships between chronological age and CBCL ratings (behaviour impairments/psychopathology) for preschool children with WS. Once again, this finding is in line with some earlier studies (Kennedy et al., 2006; Klein‐Tasman & Lee, 2017; Riby et al., 2014; Stinton et al., 2010), but not others (Dodd & Porter, 2009; Klein‐Tasman & Lee, 2017; Leyfer et al., 2006; Porter et al., 2009; Vicari et al., 2016). Similarly, no significant relationships were obtained for controls without WS.

We found no significant associations between behaviour problems/psychopathology (CBCL ratings) and overall level of developmental ability in pre-schoolers with WS, consistent with prior research with both pre-schoolers with WS (Klein‐Tasman & Lee, 2017) and older samples (Leyfer et al., 2006; Porter et al., 2009; Stinton et al., 2010). Moreover, our results showed no significant relationships between CBCL ratings (behaviour problems/psychopathology) and verbal ability in line with some other WS studies, but not others, at least in older WS samples (Braga et al., 2018; Osório et al., 2017; Porter et al., 2009). Differences in age ranges may account for the inconsistencies. In addition, nonverbal ability was not related to reported behaviour and psychological difficulties in the preschool children with WS. This outcome is not in keeping with past studies (Osório et al., 2017; Riby et al., 2014). However, Osório et al. (2017) only included older children (n = 8) in their study and Riby et al. (2014) only looked at the relationship between nonverbal ability and anxiety.

Consistent with our findings in WS children, there were no significant relationships between behaviour /psychopathology ratings and verbal ability, nonverbal ability or overall developmental ability in controls without WS.

### Clinical Implications

Findings of variability in psychopathology among preschool children with WS suggests it is important not to rely solely on group averages. Therefore, we recommend individualised assessment and management of young people with WS. The results show a high prevalence of behaviour and psychological impairments in preschool children with WS, highlighting the importance of early identification and intervention. Moreover, the distinct patterns of behaviour impairments/psychopathology (CBCL ratings) in pre-schoolers with WS and the control group must be considered when planning early interventions for these children.

Children with WS are somewhat challenging to assess at a young age given their verbal and motor delays, their reduced attention span relative to their chronological age, cognitive and physical fatigue, and often their hyper-sociability and anxiety. There are general resources that can assist researchers and clinicians with how to validly and reliably assess children with developmental delay or intellectual disability that applies also to assessing children with WS, and researchers and clinicians who are required to assess young children with WS are encouraged to seek appropriate training in test administration, gaining rapport and how to appropriately manage challenges such as inattention, anxiety or test avoidance (e.g., see Sattler, 2002). For example, observing the child for signs of fatigue, having frequent breaks, praise and positive reinforcement for effort, and remaining calm, flexible and organised are just some of many tips. It is also important to be conscious of potential floor effects, and impacts of verbal or motor delays on test validity, as well as understanding the limitations of the tests being administered.

### Limitations and Future Directions

Our study has some limitations. Firstly, the small sample size, although comparable or even larger than similar studies on WS, does raise some caution in terms of the reliability and generalisability of these exploratory study findings and group comparisons. The use of a parent-report measure to assess behaviour impairments and psychopathology in WS does not allow respondents to express themselves fully as in the case of interviews, and only provides a snapshot in time. Future studies should focus on using a combination of both informant-report measures and clinical interviews when exploring psychopathology and behaviour in WS. Moreover, the use of more than one informant would be useful, such as an educator.

Since the current study was not a longitudinal study we could not examine whether psychopathology in preschool children with WS is stable over time. It recommended that future studies use longitudinal designs; this would also allow for the identification of early predictors/risk factors for later behavioural and psychopathological impairment. Future researchers are also urged to use larger samples and to replicate our findings on other WS cohorts.

## Conclusion

Our findings highlight variations in the pattern of relationships between chronological age, and behaviour impairments/psychopathology among preschool children with WS and controls without WS, which needs to be considered in planning early interventions for these children. In addition, differences in behaviour impairments/psychopathology between older children, adolescents and adults with or without WS are also present during early childhood. Behaviour impairments/psychopathology reported in older children, adolescents and adults with WS exist during early childhood. Variability in psychopathology reported by few studies in older children, adolescents and adults with WS are, generally speaking, also found in preschool children with WS. Clinicians need to focus on developing earlier interventions to help in managing psychopathology in preschool children with WS, so as to optimise future outcomes.
